# Massive Warfarin-Induced Lingual Hematoma: A Case Report

**DOI:** 10.7759/cureus.116237

**Published:** 2026-09-14

**Authors:** Hiba Haouani, Zineb Boubekri, Sarah Hattach, Zayna Nadhil, Nadia Fellat

**Affiliations:** 1 Cardiology, Ibn Sina Hospital, Faculty of Medicine and Pharmacy of Rabat, Mohammed V University, Rabat, MAR

**Keywords:** airway obstruction, atrial fibrillation, bleeding risk, oral anticoagulant, warfarin

## Abstract

Vitamin K antagonist (VKA) therapy can cause major bleeding, most commonly GI or intracranial. Airway hematomas, though rare, are potentially life-threatening. Spontaneous lingual hematoma is an exceptionally uncommon presentation, insidious in onset and potentially rapidly fatal if overlooked. We report an elderly woman on warfarin for atrial fibrillation (AF) who developed progressive tongue swelling, dysphonia, and dyspnea over 24 hours, without trauma, a dental procedure, or fever. Examination revealed cervical and lingual swelling with gingival bleeding. Despite a clinically threatened airway, she was managed conservatively with emergent reversal using four-factor prothrombin complex concentrate and IV vitamin K, with close monitoring. Warfarin was switched to apixaban, with full recovery on follow-up. This case highlights a rare bleeding site associated with warfarin therapy, with only nine cases reported to date, and underscores the challenge of anticoagulant selection in frail, high-risk elderly patients, in whom bleeding and thrombotic risks must be individually balanced.

## Introduction

Gastrointestinal, genitourinary, and intracranial bleeding are well-recognized complications of warfarin therapy. However, airway hematoma occurring in patients with supra-therapeutic International Normalized Ratio (INR) on oral anticoagulants (OACs) represents an uncommon yet life-threatening presentation [1]. Airway involvement should be suspected in patients presenting with dysphagia, drooling, dysphonia, cervical swelling, macroglossia, or an inability to speak. Conservative management is often sufficient, but a palliative airway, such as endotracheal intubation or surgical airway, may be required.

The threatened airway makes coagulopathy reversal a necessity, which is also a strategy that inherently exposes patients to thrombotic risk determined by the underlying vitamin K antagonists (VKA) indication, including the risk of ischemic stroke in atrial fibrillation (AF) or prosthesis thrombosis in mechanical valves [2]. Herein, we report a rare case of spontaneous lingual hematoma on VKA therapy presenting as macroglossia and discuss the challenging management dilemma it raises, especially in the elderly.

## Case presentation

We report the case of an 82-year-old woman who presented to the ED with progressive tongue swelling associated with dysphonia and dyspnea. Her medical history showed uncontrolled arterial hypertension with long-term severe damage, including chronic kidney disease (CKD) and AF. She had already experienced two episodes of heart failure that were successfully treated on an outpatient basis. She was receiving the following medical treatments: beta-blockers, an angiotensin-converting enzyme inhibitor (ACEi), diuretics, and VKAs.

The patient reported progressive macroglossia and bilateral cervical swelling developing within 24 hours, followed by the development of spontaneous ecchymosis over the neck, torso, and upper limbs. No history of trauma was reported.

Physical examination showed severe inspiratory stridor and a clearly swollen, edematous tongue, visible through her slightly open mouth, which appeared to represent a large lingual hemorrhage (Figure 1). There was also gingival bleeding and diffuse anterior suprathoracic ecchymosis. The ECG showed AF, and the transthoracic echocardiogram confirmed the non-valvular AF, with slight left atrial dilatation, left ventricular hypertrophy, and a normal left ventricular ejection fraction (LVEF).

**Figure 1:**
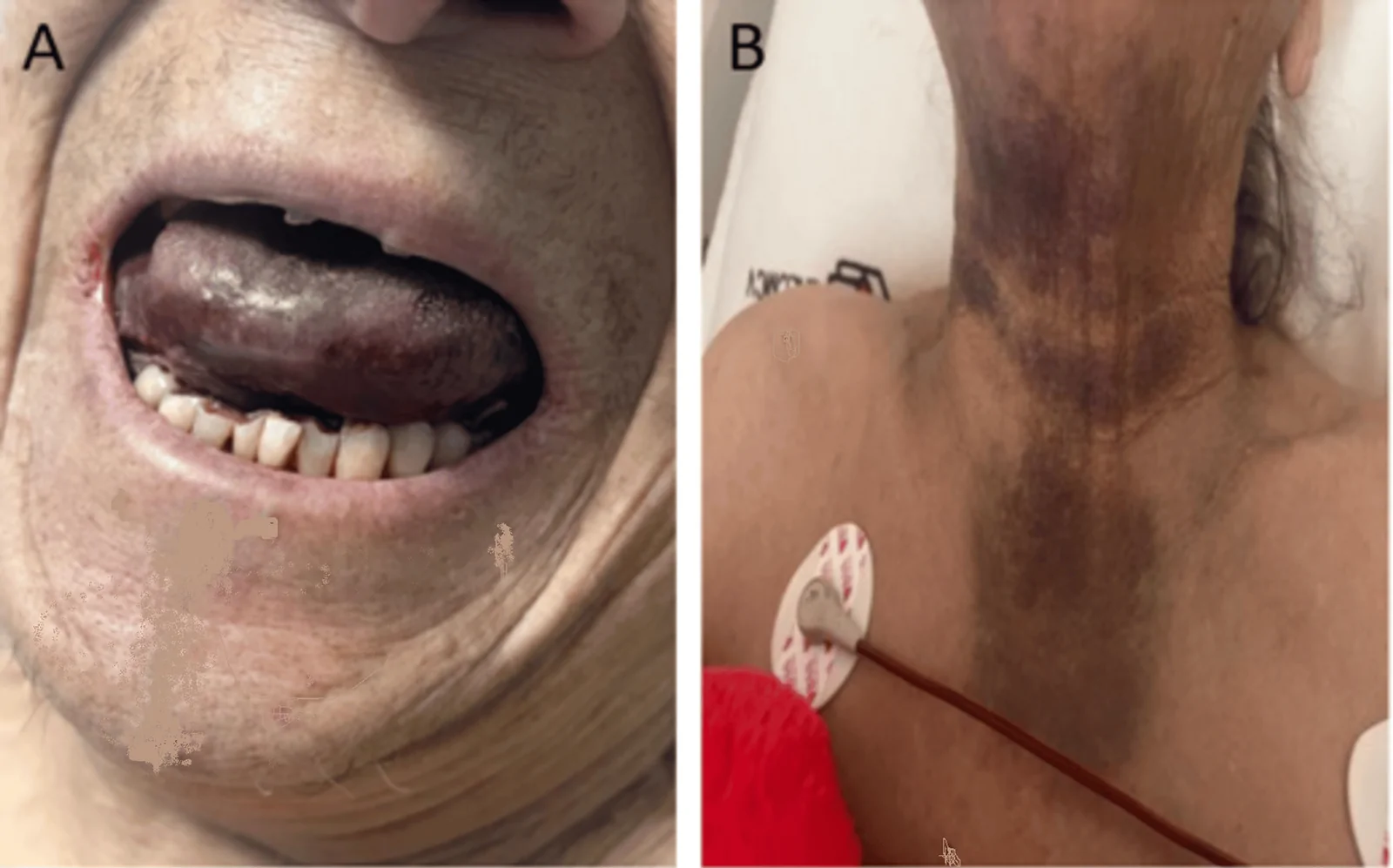
Clinical examination during the first 24 hours of coagulopathy reversal showing: (A) lingual hematoma with macroglossia; and (B) suprathoracic and bilateral cervical ecchymosis with bilateral cervical swelling.

Given the context of warfarin therapy and the absence of trauma, VKA-related major bleeding was suspected. She was evaluated by the otolaryngology service, and nasal endoscopy was performed, revealing lingual and sublingual hematoma without involvement of other upper airway structures. The patient was then admitted to the ICU for close monitoring. Biological workup showed a supra-therapeutic INR of 7, altered renal function with an estimated glomerular filtration rate (eGFR) of 38 mL/min/1.73 m², and normochromic normocytic anemia with a hemoglobin level of 10.7 g/dL, which was stable compared to baseline (Table 1).

**Table 1:** Laboratory findings on admission. INR: International normalized ratio; eGFR: Estimated glomerular filtration rate; CKD-EPI: Chronic Kidney Disease Epidemiology Collaboration; VKA: Vitamin K antagonist.

Parameter	Value	Normal Range	Interpretation
eGFR (CKD-EPI)	38 mL/min/1.73 m²	≥90 mL/min/1.73 m²	Reduced renal function
Hemoglobin	10.7 g/dL	12-15.5 g/dL	Anemia, stable compared to baseline
INR	7	0.8-1.2 (therapeutic range 2.0-3.0 if on VKA)	Supratherapeutic

The diagnosis of severe VKA over-anticoagulation was confirmed. Management consisted of immediate coagulopathy reversal according to current recommendations: four-factor prothrombin complex concentrate (50 IU/kg IV) and 10 mg IV vitamin K. Follow-up INR decreased to 1.26. Antibiotics for anaerobic coverage were also administered, along with corticosteroids to reduce edema and soft tissue infiltration.

The patient remained hemodynamically stable, with stable respiratory status. Airway intervention was fortunately not required. Progressive regression of the lingual and cervical hematoma was observed (Figure 2). After five days, anticoagulation was restarted with apixaban, and she was discharged in stable condition. At follow-up, she had fully recovered speech and oral intake.

**Figure 2:**
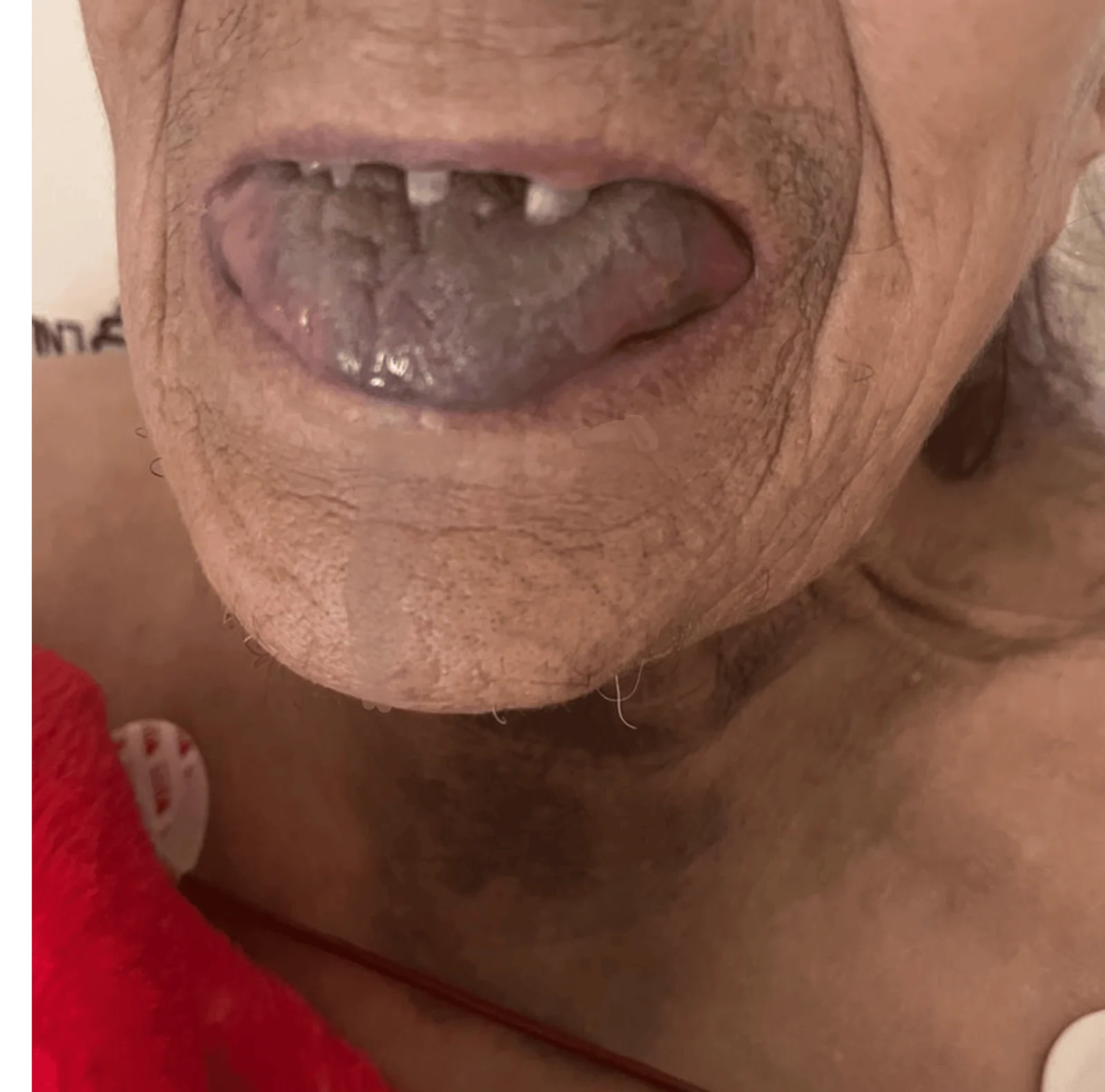
Clinical evolution after 48 hours of coagulopathy reversal showing regression of the lingual hematoma.

## Discussion

Lingual hematoma is an extremely rare manifestation of VKA-related bleeding. Although warfarin-associated hemorrhage most commonly involves the gastrointestinal tract or intracranial space, unusual soft tissue localizations may occur and can be life-threatening due to the risk of airway obstruction.

In our case, severe over-anticoagulation led to spontaneous massive tongue hematoma and cervical soft tissue bleeding, mimicking a Ludwig angina presentation. The tongue is highly vascularized via branches of the lingual artery, and intramuscular bleeding can expand rapidly within confined fascial planes, explaining the risk of sudden airway compromise. CKD and advanced age also likely contributed to increased bleeding susceptibility. The major clinical concern in lingual hematoma is rapid progression toward upper airway compromise. Early recognition of stridor and cervical swelling is essential, as delayed management may necessitate intubation or emergency tracheotomy.

Fewer than 32 well-documented airway hematoma cases related to warfarin therapy have been reported in the literature, with sublingual bleeding representing the most common airway site in this context. However, to the best of our knowledge, based on a PubMed search using the terms "lingual hematoma" and "warfarin," and after excluding duplicates, only nine cases of lingual hematoma were reported [1, 3-9]. Most cases were observed with warfarin use and were more common in the elderly. Only one case was reported in a 41-year-old patient in the context of a concomitant upper respiratory tract infection while on warfarin for a mechanical mitral valve [9].

Current recommendations from the American College of Cardiology support immediate reversal of VKA in cases of major bleeding using intravenous vitamin K and four-factor prothrombin complex concentrate (4F-PCC). Our management followed these recommendations, allowing rapid INR normalization and avoidance of airway intervention [2].

The management pathway starts with identifying the bleeding site and assessing bleeding severity. This is an initial determining step that guides management. Bleeding is considered major when it causes hemodynamic instability, when it is overt and causes hemoglobin levels to decrease by at least 2 g/dL, or when it requires transfusion of at least two units of RBCs. Bleeding at a critical site is also considered major. A list of critical sites is provided by the expert consensus paper and includes bleeding that compromises organ function and poses an imminent threat to life. In this context, upper airway hemorrhage, such as lingual and retropharyngeal hematomas, represents bleeding in anatomically dangerous soft tissue spaces where hemorrhage can progress rapidly near a vital region, justifying its classification as a major bleeding event warranting urgent reversal [2].

Bleeding assessed as major justifies discontinuation of anticoagulant therapy and, when appropriate, antiplatelet therapy, along with securing the airway. For patients on warfarin, a repletion strategy using 4F-PCC, or plasma if 4F-PCC is not available, is recommended. Vitamin K (1 to 10 mg), preferably administered as a slow IV infusion, restores hepatic carboxylation of vitamin K-dependent coagulation factors [2].

Following bleeding control, the question of anticoagulation resumption inevitably arises and constitutes the second arm of the management dilemma. Reassessment of the indication for anticoagulation is necessary. When the indication is endorsed by clinical practice guidelines, restarting anticoagulation is of net benefit, but questions often arise regarding the timing, especially if thrombotic risk is high. Elements that increase thrombotic risk include a recent stroke within the last six months, mechanical valve prosthesis, AF with a CHA2DS2-VASc score of ≥4, ischemic stroke within the last three months, and left atrial thrombus. On the other hand, bleeding characteristics such as critical location, source of bleeding (identified or not), bleeding mechanism (traumatic or spontaneous), and the need for surgical procedures are all factors that contribute to decision-making regarding when to restart anticoagulation. The decision should be discussed within a multidisciplinary team. Generally, early OAC reinstitution is favored once bleeding is controlled and the patient is clinically stable, with close monitoring. Switching to a different OAC, such as replacing a VKA with a direct oral anticoagulant (DOAC) when possible, should be considered [2,10].

Beyond the immediate management, this case raises an important broader question: should anticoagulation be continued with warfarin, or is this an opportunity to switch to a DOAC? Our patient's frailty, advanced age, comorbidities, and severe over-anticoagulation all warrant this reassessment. Her Groningen Frailty Indicator score of 6 classified her as frail, a profile associated with multiple overlapping bleeding risk factors including polypharmacy, uncontrolled hypertension, and increased fall risk: all are modifiable factors that require optimization regardless of OAC choice [10].

However, the evidence on switching OAC in frail patients is nuanced. In literature, there is limited data on switching OAC in older patients (≥75 years) with polypharmacy or other markers of frailty. The FRAIL-AF, a randomized controlled trial, that analyzed major and clinically relevant bleeding events in frail patients with AF, demonstrated that more bleeding complications were observed when switching from VKA treatment to a DOAC, compared to continuing VKA treatment. Frailty was defined based on Groningen Frailty Indicator score as superior to three [11]. This higher bleeding risk with non-VKA oral anticoagulants was not offset by a reduction in thromboembolic events, as switch to DOACs didn’t seem to decrease the risk of thromboembolic events, this risk was low in both frail groups. However, when severe bleeding occurs, with unstable or variant INR or when patient adherence is suspected, it is recommended to switch to DOAC when eligible [10].

Our case highlights the importance of strict INR monitoring in elderly patients and the potential advantage of DOACs in frail patients particularly when associated with CKD. A meta-analysis published in 2022, integrating thirty studies analyzing data of patients with AF and CKD (defined as creatinine clearance below 60 mL/min) showed that DOACs were safer and more effective than warfarin regardless of severity of renal impairment. It should be noted that renal function across the included studies was evaluated using either creatinine clearance or eGFR with no applied exclusion criteria regarding the used eGFR estimation equation. Apixaban and edoxaban presented better efficacy and safety, compared to other DOACs as well as warfarin. When creatinine clearance is below 30 mL/min, apixaban seems to be the preferred agent especially in terms of safety [12].

## Conclusions

This case illustrates an underrecognized bleeding site where the urgency of coagulopathy reversal must be carefully weighed against the thrombotic risk inherent to the underlying indication for VKA therapy. Airway status, INR level, and thrombotic risk profile should all inform this individualized, multidisciplinary decision.

Beyond acute management, this case highlights the importance of reassessing the long-term anticoagulation strategy, particularly in elderly patients with CKD. However, the decision to switch to a DOAC should not be regarded as universally beneficial. Anticoagulation strategy should therefore be individualized, guided by frailty status, renal function, and bleeding versus thrombotic risk, rather than defaulting to a DOAC switch based on the assumption of a universal safety benefit.
